# Development of a robotic-assisted handling and manipulation system for the high-scale bioproduction of 3D-bioprinted organ-on-a-chip devices

**DOI:** 10.1016/j.ohx.2024.e00572

**Published:** 2024-08-22

**Authors:** Nils Lindner, Andres Mejia-Wille, Anna Fritschen, Andreas Blaeser

**Affiliations:** a BioMedical Printing Technology, Department of Mechanical Engineering, Technical University of Darmstadt, Germany; b Centre for Synthetic Biology, Technical University of Darmstadt, Germany

**Keywords:** Lab automation, Industrial translation, Biofabrication, Organs-on-a-chip, OoC, Demonstrator

## Abstract

Organs-on-a-chip (OoCs) have proven to mimic the basic physiological behavior of organs and the influence of therapeutics on them in greater detail than conventional models, resulting in enormous projected market growth rates. However, the breakthrough to profitable commercialization of that technology has not yet been achieved, partly because the production process chain is characterized by a high proportion of manual laboratory work. The present work addresses this point. Utilizing affordable components, a demonstrator was developed that can be integrated into an existing 3D-bioprinting system and enables the automated production of perfusion-ready OoC devices starting from pre-fabricated injection-molded microfluidic chips. To this end, a corresponding process chain was first defined, and an expandable, configurable algorithm was developed and validated in the form of a finite state machine (FSM). This algorithm controls a modified 4-axis robot arm that covers the steps upstream and downstream of the printing process in the manufacturing process and achieves success rates of up to 100 %. A virtual interface between the robot and printer enables mutual communication and full integration of the algorithm into the process chain. Steps that pose a challenge for the automation of the process chain and appropriate countermeasures and optimizations were identified. This lays the foundation for scaling and standardizing the automated production of OoCs.

## Graphical abstract

**Figure:**
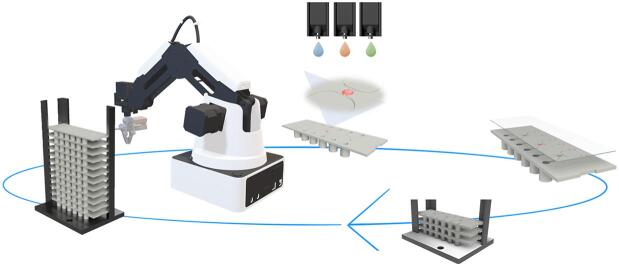

Specifications table

**Table:** 

Hardware name	• robotic-assisted handling and manipulation system for high-scale bioproduction of OoCs
Subject area	• Engineering • Medical (e.g., pharmaceutical science) • Biological sciences (e.g., microbiology and biochemistry) • Educational tools and open-source alternatives to existing infrastructure
Hardware type	• Biological sample handling and preparation Electrical engineering and computer scienceMechanical engineering and materials science
Closest commercial analog	No commercial analog is available
Open source license	CC BY 4.0
Cost of hardware	1618.28 €
Source file repository	https://doi.org/10.17632/jm4ndf8r9j.1

## 1 Hardware in context

**Fig. 1:**
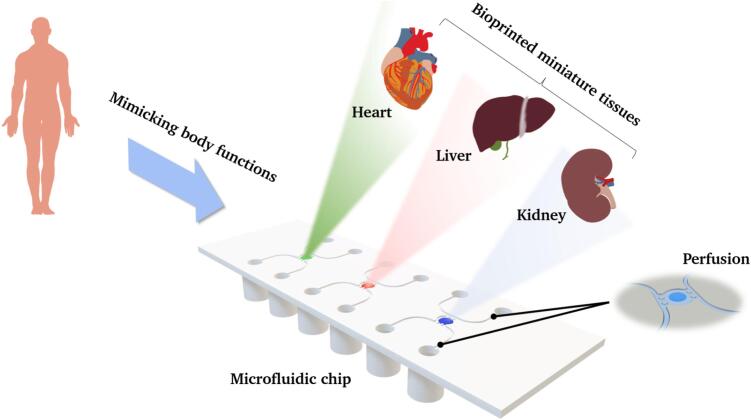
Schematic illustration of a microfluidic chip with three reaction chambers filled with different cell types as a body-on-a-chip (BoC) model with perfusable channels.

The fabrication of so-called organs-on-a-chip (OoCs) is part of ongoing research, and the development of such systems is increasingly attracting clinical and industrial interest [1], [2]. OoCs are microfluidic platforms that are used to replicate (partial) functions as well as physiological and mechanical properties of individual or multiple organs in 3D cell culture to mimic human biological processes and, for example, to better understand or test the effects of drugs on the human body [3], [4]. Therefore, one or more three-dimensional reaction chambers are located on these chips, which can be interconnected via channels and supported by medium in order to simulate a dynamic environment and enable perfusion. The reaction chambers can be filled with cells or cell-laden bio-inks to grow functional tissue units (Fig. 1).

Despite the high level of interdisciplinarity and the resulting complexity, OoCs already have a fairly high technology readiness level (TRL) compared to the considerably more complex fabrication and cultivation of larger organ patches or even whole organs. Research groups with versatile expertise increasingly achieve success in terms of suitable biomaterials and their processing as well as biochemical functionality and transferability of the models [5], [6], [7] so that a rising number of possible applications can be addressed. The range of applications usually refers to toxicological and pharmaceutical research [8], drug discovery and development [9] and personalized medicine [10]. The broad utility, advantages over traditional 2D models, and the prospect of potentially eliminating animal testing in the long run [11] show the vast potential behind the technology and the disruptive impact it can and will have on society as a whole.

**Fig. 2:**
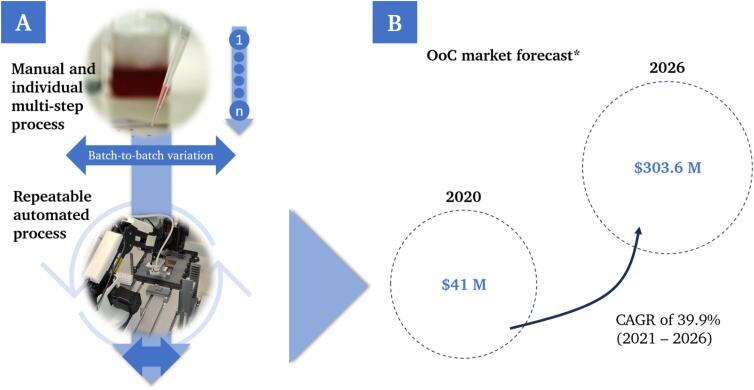
Shift from a multi-step and manual process in the laboratory to an automated and repeatable process chain (A) as a prerequisite for the predicted commercial success (B). *Estimation of market growth for OoCs based on Compound Annual Growth Rate (CAGR) [14].

In addition to the biological and technical implementation, production and the associated process chain have an important role to the successful clinical and industrial translation of OoCs. Alongside the major impact in terms of possible applications and the associated effects on a healthy society or advances in ethical animal welfare, enormous economic potential is also inherent in the technology [12], [13]. Current approaches are usually produced on a small scale in laboratories at research institutions and vary greatly from batch to batch due to the influence of many manual steps and the respective user (Fig. 2A). So far, standardized and automated manufacturing is not considered much in this context, although this should be given early attention and be one of the next logical steps. Not only can this save personnel, time, and costs, but an automated and standardized implementation can also have a positive effect on successful approval of drugs and medication, given the strict rules and regulations in pharmaceutical research. A transfer from the current laboratory application to a larger-scale automated production is necessary to reach the predicted market potential of OoCs (Fig. 2B). Current approaches, such as the BioAssembly Platform (Advanced Solutions Life Sciences) as an integrated biofabrication platform, involve high investment costs and require experienced users to implement planned projects. Other solutions for automated assays, such as the HUMIMIC AutoLab (TissUse GmbH), only cover the cultivation and evaluation phase.

In the specific context of OoC production in combination with 3D-bioprinting^1^, up-scaling, automation and regulatory hurdles are often still seen as bottlenecks [15], [16] to exploit the highly perceived economic potential behind the technology [14]. Laboratory automation in general is a broad topic with versatile aspects, which has surrounded research and translation for years. Numerous successful examples exist that are widely applied [17], [18], [19]. However, no commercial approaches, especially those based on open source hardware, exist for the specific use case to automate the production of OoCs. This is due to the novelty of OoC- and 3D-bioprinting-technology itself, dependence of the process on the chip used, existing laboratory infrastructure and individual experience of the user.

**Fig. 3:**
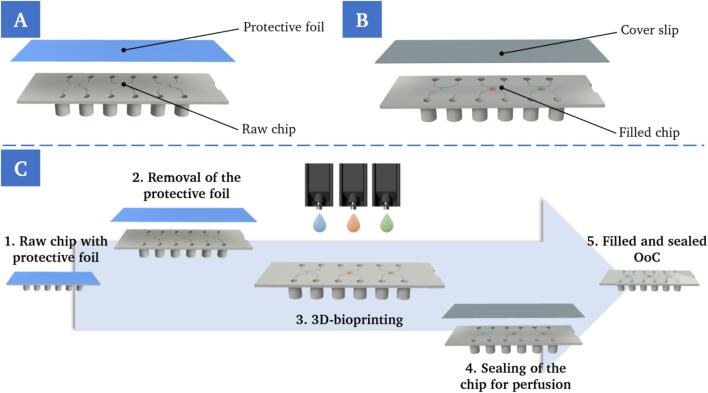
Exploded view of the components of the unprocessed chip in its initial state, consisting of the chip itself and a protective foil (A). Exploded view of the filled and sealed chip, consisting of the chip, that was filled with bio-ink by the 3D-bioprinter, and a transparent cover slip for tight seali ng (B). Schematic representation of the states and necessary basic process steps for the production of OoCs, based on the selected chip design (C).

This work addresses the automated transport, manipulation of and printing on existing microfluidic chips and thus fits in between the fabrication of the raw microfluidic chip and the maturation of small functional tissue models. Explicitly, a reliable process chain was developed with a repeatable and standardized workflow and implemented into an existing 3D-bioprinting system. The process area addressed here ranges from a pre-fabricated injection molded microfluidic device to a tightly closed and perfusion-ready OoC device. Whenever the degree of automation of the process chain is discussed in this paper, it always refers to this defined process area. Integration into a closed system also allows work in a sterile environment. Sterility of OoCs is, similar to conventional cell culture platforms, crucial to avoid microbial contamination. During the development, the focus was on feasibility and low investment costs, but at the same time reliability and transferability for industrial environments. Prior to the implementation of the overall system, it was first necessary to identify a concept. Individual process steps were identified based on an existing chip design, followed by the assembly of a process chain and identification of the required components for implementation. In general, the process chain (Fig. 3C) is highly dependent on the utilized chip. For the application shown here, a custom-made microfluidic chip from ibidi (ibidi GmbH, Graefelfing, Germany), with standard slide format according to ISO 8037/1 and female Luer connectors, was used. The chip is initially sealed with a protective foil to prevent contamination (Fig. 3A), that must be removed before printing. After printing on the chip, it is sealed with a COP cover slip to subsequently enable perfusion (Fig. 3B). The bioprinter used was a microvalve-based drop-on-demand (DOD) bioprinter (SuperFill, Black Drop Bioprinter GmbH, Aachen, Germany) with nozzle diameters of 300 and 150 µm. With microvalve-based DOD-bioprinting in general, individual droplets with volumes between 2 and 720 nl can be dispensed from a pressurized fluid reservoir with high precision by opening and closing the valve [20].

**Fig. 4:**
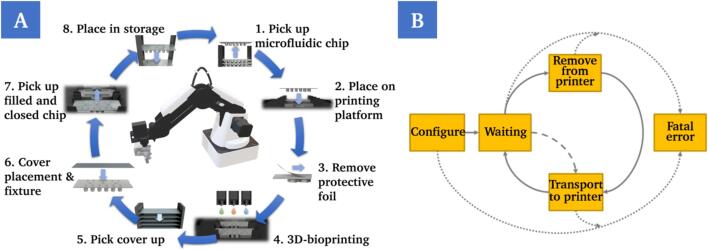
Schematic illustration of the process chain for the repeatable and automated production of OoCs based on the chosen system and chip design (A). Schematic representation of the logic behind the implementation of the system as a finite state machine (B).

For the automated production of multiple OoCs in a closed system, based on the chip design used, further intermediate steps and components must be added. A robot with a two-finger gripper as an end–effector was used to manipulate the chips. Supply and storage points as well as holders must also be added. The basic process chain (Fig. 4A) contains eight steps and is repeatable. The microfluid chips must be removed from a supply with the aid of the robot (1), delivered to the printing platform and placed on a fixture (2). At this position, the microfluidic chip is filled with the cell-containing bio-ink by the 3D-bioprinter. Before this can happen, a protective foil that is covering the chip to prevent contamination with bacteria must be removed and disposed of (3). The robot must then leave the printing area and wait until the printing process is complete in order to avoid collisions and thus damage to the system (4).

After printing is complete, the microfluidic chip must be sealed tightly to ensure perfusion. According to this, the robot must convey a transparent cover slip from another supply to the printing area (5), place it precisely on the filled microfluidic chip, center and fix it in place (6). Finally, the filled microfluidic chip is removed from the fixture (7) and transported to the storage area (8). The process is repeatable and enables the automatic production of multiple chips in sequence. The developed process chain is optimized for the chip design used, based on standard microscope slides and an adhesive bonding between chip and cover slip. Other approaches, such as those that ensure sealing for perfusion via a plug-in or screw mechanism, are not compatible with the system described here. According to the SiLA consortium (Association Consortium Standatization in Lab Automation), the system was implemented as a finite-state machine (FSM) (Fig. 4B) and thus allows the developer to logically structure the developed algorithm and to explicitly define state changes so that the process is always in a defined frame with defined states.

The long-term potential for cost reduction through the use of processes and methods for laboratory automation is high, but often associated with high acquisition costs and down-times. This discourages users from making these investments, especially for new processes such as the automated production of OoCs. For this reason, it is even more important to thoroughly understand such a process before automating it, to coordinate and harmonize the process steps involved with each other and to validate the functionality. In this work, a demonstrator for the realization of the described task was successfully built (see chapter 5), brought to operation (see chapter 6) and validated (see chapter 7). The presented system is easy to adapt, customizable and allows a transfer to the use in research laboratories as well as for high-scale use in industry.

## 2 Hardware description

A modified educational robot, sensors, a microcontroller, and 3D-printed parts were used to develop a system that is capable to place, manipulate, and store samples in a 3D-bioprinter to pave the way for automated OoC production. In addition, an interface between robotics and 3D-bioprinter was created to allow communication between the two systems.

### 2.1 Overall design

**Fig. 5:**
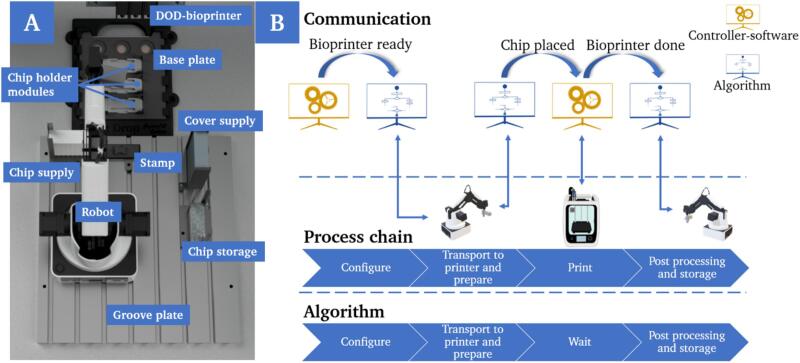
Rendering of the assembly in the working area of the bioprinter from top-view. The setup comprises the 4-axis robot including sensors and the bioprinter itself as active components. A chip supply, chip holder, stamp, coverslip supply, and chip storage are mounted as passive components (A). Communication between the manipulation system and the bioprinter was realized via an open interface. The transfer of data between the controller software of the bioprinter and the developed algorithm is emulated via a virtual COM port and the communication and logic behind the controller is implemented via a Python script (B).

Throughout the development process, the aim was to find a flexible and at the same time compact solution that is easy to modify for the specific application and preexisting infrastructure. The entire process chain and the associated components were integrated into an existing bioprinting system with a sterile hood as embodiment and robotics based on a modified CNC milling machine. The physical structure (Fig. 5A) as well as the associated control system and underlying logic (Fig. 5B) must both be considered.

The setup is designed to be compact to ensure that the working area of the selected robot is large enough to reach all stations and that pre-assembly on a compact groove plate is possible. Several advantages arise from that. On the one hand, the space-saving design reduces the required build area, and the overall system can even be integrated into smaller work cells. On the other hand, the modular design makes it easy to remove and insert all the required components at the same time, without the need for repeated recalibration, thus allowing flexible use of the 3D-bioprinter. This eliminates the need for the printer to be used exclusively for this specific application and avoids time-consuming set-up and conversion work. The developed process chain in general is universally applicable and must only be adapted to external factors such as the substrate or chip used. The passive structure (in particular supply stations, holders and storages) is conceptualized and configured for the specific chip design used in this work. However, these components can be easily adapted in terms of design and can be flexibly manufactured for any other specific chip design using 3D-printing.

A virtual interface between the robot and the bioprinter was created to facilitate the transferability of the solution and make it independent of the bioprinting system used and the corresponding software. The underlying logic was implemented in a Python script. A configuration file is loaded before the script is executed so that adjustments to the physical structure can also be implemented quickly in the control system. This configuration file contains predefined values, which include the position of the respective stations and the geometry of the chip used. The manufacturer’s Python API is used to control the manipulation robot. The software of the bioprinter and the script for operating the robot exchange in- and output signals bidirectionally via the virtual serial interface (Com0Com), whereby the output of one port is always the input of the second port.

However, if the hardware or software of the selected printing system does not offer the option of processing signals via a serial interface, a procedure in which the printing process and handling are started sequentially by the operator is also feasible. This would eliminate the benefit in terms of time due to the parallelization of work steps in the laboratory but would still prevent extended intervention by the user in the sterile working environment.

As an alternative solution to using the manufacturer’s own Python API and implementing the logic for the overall system via a Python script, the Robot Operating System (ROS) framework can be mentioned, which would for example allow the exchange of messages between programs that are executed simultaneously. Since this has no influence on the way in which data is extracted from the printer software and further processed, this approach was not pursued further in this study. The same applies to other frameworks such as MQTT, OPC-UA and DDS. Another major advantage of the developed solution is its simplicity and adaptability. Learning the ropes of such frameworks can sometimes be tedious and require experience. The chosen approach only requires rudimentary knowledge of the Python language and how to import pre-built functions via the Python API.

The individual elements and components that comprise the system are briefly explained in more detail below.

### 2.2 Dobot magician

The robot used is a 4-axis Dobot-Magician (C1) from Shenzhen Yuejiang Technology Co, Ltd [21], which is marketed as a small desktop robot arm for educational purposes. The interchangeable end-effector allows the use of self-developed solutions. Furthermore, the support of different programming languages, e.g., C# and Python, contributes to the versatility of the system. The robot has multiple interfaces through which sensors and actuators can be connected. Moreover, the robot is well suited for this project because its compact and lightweight design allows it to be placed in the bioprinter's workspace.

A simple two-finger parallel gripper is used as an end-effector for handling and manipulating the samples, which applies its force pneumatically via a connected pump. To improve functionality and process stability, the gripper design was optimized and adapted for the specific application and configuration (see 2.4.1).

### 2.3 Sensors

The simple monitoring of critical process phases is reduced to the use of only a few sensors and simple methods for data processing, still covering all critical aspects.

#### 2.3.1 Ultrasonic and temperature sensor for distance measurement

The primary reason for integrating an ultrasonic sensor is to increase system reliability. To illustrate this, the removal of a microfluidic chip from the supply can be considered. Assuming that the algorithm works without sensor data, the position at which the gripper is moved for removal must be specified for each cycle. This can be achieved, for example, by passing the initial quantity of stacked microfluidic chips and the height of a single chip to the algorithm. Although this solution is easy to implement, it is sensitive to changes. For example, if the user specifies an incorrect number of microfluidic chips or if the algorithm is restarted and the current number of stacked microfluidic chips is not updated. In such cases, the algorithm would not notice the changes and would continue to run, leading to errors. Therefore, an ultrasonic sensor (S1) was integrated, that measures the distance between gripper and the samples to be picked up. An ultrasonic sensor was chosen over alternatives because of the transparent cover slip, which is placed on the microfluidic chip after the printing process. Laser sensors are unsuitable for this application, as they cannot determine the distance to a transparent target, while the color and transparency of the target object have no influence on the measured distance with an ultrasonic sensor.

Considering that the propagation speed of sound in air depends on the surrounding temperature, a temperature sensor (S2) is attached to the robot to determine the current room temperature and to calculate the adjusted propagation speed based on this.

#### 2.3.2 Camera for monitoring the removal of the protective foil

A critical process step that can lead to errors is the removal of the protective foil from the chip before printing. As this step must be completed successfully before the subsequent process steps can be performed, a monitoring function has been implemented. A defined Region of Interest (ROI) is recorded via a camera (S3) that is directed at the printing platform from top. The brightness value returned for the defined area (position of the respective chips) is used to determine whether the protective foil was peeled off successfully. If this step is not successful, the removal of the foil is repeated. Unlike the previously presented sensors, the camera is directly connected to the central processing unit via USB.

#### 2.3.3 Microcontroller

An Arduino Mega 2560 Rev3 (C2) is used to read out and pass on the sensor data. The board not only collects and processes the sensor data, but also supplies the sensors on the robot with power.

### 2.4 3D-printed parts

To allow flexible and quick adjustments to the gripper or other components, the majority of the parts used were 3D-printed (with a Bambu Lab X1 Carbon FFF-printer and a 0.4 mm nozzle). Standard PLA was used as the material for all printed parts. The 3D-printed parts are briefly described below.

#### 2.4.1 Gripper

The original gripper of the selected robot is not optimally suited for removing the protective foil from the chips used in this work. To account for this, a gripper optimized for the task was designed (P9 – P14). This is based on the original geometry but is adapted in terms of surface angle and width.

#### 2.4.2 Platform module and sample holder

A fixed position on the printing platform is provided for the insertion of sample holders or corresponding modules. The base plate (P3) for this specific application has a flexible design and allows three modules (P17–P19) to be inserted at the same time into press fits. These can be designed according to individual requirements and serve as sample holders for the chips to be filled by the bioprinter. If, for example, the chips need to be temperature controlled during printing, thermally conductive material can be used. In addition, the holder for the chips should be selected so that the chips automatically lock into place and fit tightly enough that they do not come loose from the holder when the protective foil is removed. At the same time the chips must be loose enough to be gripped and removed by the robot afterwards. The designed base plate also supports the centered placement of the respective cover slips to close the chips after printing.

#### 2.4.3 Supply and storage

Various supply and storage stations are required for the repeatable execution of the process chain. This comprises a supply system (P22) for the raw, unfilled microfluidic chips, a supply system for the cover slips (P6) and a storage system for the filled and sealed chips (P22). The supply of empty chips and the storage for filled chips are identical.

#### 2.4.4 Stamp

To ensure that the filled microfluidic chip is sealed and to enable perfusion during subsequent application, a stamp is used to fix the cover slip after it has been placed. To do so, the robot must pick up the stamp (P20) from its dedicated position and press it onto the foil and the chip with a defined force. The stamp is designed in such way that it fully contacts the cover. With the next step, the robot returns the stamp to the stamp rack (P21), picks up the closed microfluidic chip and transports it to storage.

#### 2.4.5 Auxiliary constructions

Additional components were produced using 3D-printing to attach the applied sensors. This includes a mount for the ultrasonic sensor (P15–P16), the temperature sensor (P7–P8) and the microcontroller (P1–P2) directly on the robot as well as a mount for the camera (P4–P5), which can be attached above the system.

### 2.5 Advantages and utilization

No comparable commercial solution exists to date that is explicitly designed for the manipulation of substrates in the production of OoCs in combination with 3D-bioprinting and is integrated into the associated process chain. It should also be underlined that the costs were kept low for easy access. The simplicity and adaptability of the system should also lower the reluctance to adapt it in other laboratories.

In addition to the simple automation of the manual handling steps, the system can also allow the parallelization of process steps. This allows empty chips to be brought to the printing platform or already loaded chips to be removed while chips are being filled with bio-ink by the bioprinter simultaneously. The implementation as an FSM is in line with laboratory standards and forms a solid basis for a standardized and safe production process.

Additional planar axes can be added to the system to move workstations or the manipulation system, for instance, to create an even more flexible environment and thus the basis for networked and adaptive production. In general, further tasks involving the manipulation and movement of laboratory samples in a defined work area can be performed by the system through simple adaptations.

The following bullet points briefly summarize the benefits and potential applications of the system and how it can impact standard or novel laboratory tasks:

• Automation of intermediate steps in 3D-bioprinting.
• Standardized and repeatable approach that is easy to embed in a sterile working environment.
• Reliable automated manipulation of laboratory samples (in this case microfluidic chips).
• Cost-effective access to laboratory automation.
• Easy adaptability to specific other applications.

***Design files***

## 3 Design files summary

The .stl files associated with the respective CAD files for 3D-printing the parts listed above can be found under the link to the repository (Table 1).

**Table 1:** Design file summary.

Design file name	File type	Open source license	Location of the file
D1_ArduinoHolder_prt1.ipt	CAD	CC BY 4.0	https://doi.org/10.17632/jm4ndf8r9j.1
D2_ArduinoHolder_prt2.ipt	CAD	CC BY 4.0	https://doi.org/10.17632/jm4ndf8r9j.1
D3_BasePlate.ipt	CAD	CC BY 4.0	https://doi.org/10.17632/jm4ndf8r9j.1
D4_CamHolder_prt1.ipt	CAD	CC BY 4.0	https://doi.org/10.17632/jm4ndf8r9j.1
D5_CamHolder_prt2.ipt	CAD	CC BY 4.0	https://doi.org/10.17632/jm4ndf8r9j.1
D6_CoverSupply.ipt	CAD	CC BY 4.0	https://doi.org/10.17632/jm4ndf8r9j.1
D7_DHT11Holder_prt1.ipt	CAD	CC BY 4.0	https://doi.org/10.17632/jm4ndf8r9j.1
D8_DHT11Holder_prt2.ipt	CAD	CC BY 4.0	https://doi.org/10.17632/jm4ndf8r9j.1
D9_Gripperleft_8deg.ipt	CAD	CC BY 4.0	https://doi.org/10.17632/jm4ndf8r9j.1
D10_Gripperright_8Deg.ipt	CAD	CC BY 4.0	https://doi.org/10.17632/jm4ndf8r9j.1
D11_Gripperleft_10deg.ipt	CAD	CC BY 4.0	https://doi.org/10.17632/jm4ndf8r9j.1
D12_Gripperright_10deg.ipt	CAD	CC BY 4.0	https://doi.org/10.17632/jm4ndf8r9j.1
D13_Gripperleft_15deg.ipt	CAD	CC BY 4.0	https://doi.org/10.17632/jm4ndf8r9j.1
D14_Gripperright_15deg.ipt	CAD	CC BY 4.0	https://doi.org/10.17632/jm4ndf8r9j.1
D15_HC-SR04Holder_prt1.ipt	CAD	CC BY 4.0	https://doi.org/10.17632/jm4ndf8r9j.1
D16_HC-SR04Holder_prt2.ipt	CAD	CC BY 4.0	https://doi.org/10.17632/jm4ndf8r9j.1
D17_Module1.ipt	CAD	CC BY 4.0	https://doi.org/10.17632/jm4ndf8r9j.1
D18_Module2.ipt	CAD	CC BY 4.0	https://doi.org/10.17632/jm4ndf8r9j.1
D19_Module3.ipt	CAD	CC BY 4.0	https://doi.org/10.17632/jm4ndf8r9j.1
D20_Stamp.ipt	CAD	CC BY 4.0	https://doi.org/10.17632/jm4ndf8r9j.1
D21_StampRack.ipt	CAD	CC BY 4.0	https://doi.org/10.17632/jm4ndf8r9j.1
D22_SupplyStorage.ipt	CAD	CC BY 4.0	https://doi.org/10.17632/jm4ndf8r9j.1
video1.mp4	video	CC BY 4.0	Available with the article
video12.mp4	video	CC BY 4.0	Available with the article
video3.mp4	video	CC BY 4.0	Available with the article
video4.mp4	video	CC BY 4.0	Available with the article

***Bill of materials***

## 4 Bill of materials summary

**Table 2:** Bill of materials (cost in €).

Designator	Component	Number	Cost per unit-currency	Total cost-currency	Source of materials	Material type
C1_robot	Dobot Magician V2	1	1363.78 €	1363.78 €	https://www.reichelt.de/roboterarm-dobot-magician-advanced-dobot-magician-a-p249871.html?&nbc=1&trstct=lsbght_sldr::249872	Robot
C2_microcontroller	Arduino Mega 2560 Rev3	1	42.00 €	42.00 €	https://store.arduino.cc/products/arduino-mega-2560-rev3	Microcontroller
C3_groove plate	Isel T-Slot-Plate PT50 (B 375 mm x H 20 mm x L 400 mm)	1	118.76 €	118.76 €	https://www.isel.com/de/t-slot-plates-pt50.html	Aluminium
C4_screws M6 x 20 mm	Screws (M6 x 20 mm)	12	1.01 €	12.12 €	https://www.eisenwaren2000.de/Zylinderschrauben-M6-X-20-mit-Innensechskant-DIN-912-Edelstahl-A2	Stainless steel
C5_screws M6 x 50 mm	Screws (M6 x 50 mm)	4	1.13 €	4.52 €	https://www.eisenwaren2000.de/Zylinderschrauben-M6-X-50-mit-Innensechskant-DIN-912-Edelstahl-A2	Stainless steel
C6_sliding blocks M6	Sliding Blocks (M6)	12	1.12 €	13.44 €	https://www.isel.com/de/t-nutensteine-m6-ve-20-stuck-fur-pt-re-40–65.html	Steel
S1_Ultrasonic Sensor	HC – SR04	1	3.80 €	3.80 €	DEBO SEN ULTRA: Entwicklerboards – Ultraschall Abstandssensor, HC-SR04 bei reichelt elektronik	Sensor
S2_temperature sensor	DHT11	1	1.84 €	1.84 €	DHT11 digitaler Feuchtigkeits- und Temperatursensor | Roboter-Bausatz.de	Sensor
S3_webcam	Genius WideCam F100 V2	1	46.70 €	46.70 €	GENIUS WideCam F100 V2/Full-HD 1080P/ USB/ | Kaufland.de	Sensor
Filament	PLA NX-2	435.49 g	25.99 €/kg	11.32 €	https://www.3djake.de/extrudr/pla-nx-2-neon-orange?sai=9495&gclid=CjwKCAiAkp6tBhB5EiwANTCx1OviiLEWBRtPmVub7YhrQHxHfT7uKM0Fln2Nq3gF6oKW-XjccNExcBoCe7cQAvD_BwE	PLA
P1_ArduinoHolder_prt1.stl	arduino-holder	31.16 g	0.81 €	0.81 €		PLA
P2_ArduinoHolder_prt2.stl		7.10 g	0.18 €	0.18 €		PLA
P3_BasePlate.stl	base plate	95.96 g	2.49 €	2.49 €		PLA
P4_CamHolder_prt1.stl	cam-holder	6.77 g	0.18 €	0.18 €		PLA
P5_CamHolder_prt2.stl		8.87 g	0.23 €	0.46 €		PLA
P6_CoverSupply.stl	cover supply	54.88 g	1.42 €	1.42 €		PLA
P7_DHT11Holder_prt1.stl	DHT11-holder	6.54 g	0.17 €	0.17 €		PLA
P8_DHT11Holder_prt2.stl		2.89 g	0.08 €	0.08 €		PLA
P9_Gripperleft_8deg.stl	gripper_8deg	6.30 g	0.16 €	0.16 €		PLA
P10_Gripperright_8Deg.stl		7.07 g	0.18 €	0.18 €		PLA
P11_Gripperleft_10deg.stl	gripper_10deg	5.39 g	0.14 €	0.14 €		PLA
P12_Gripperright_10deg.stl		6.83 g	0.18 €	0.18 €		PLA
P13_Gripperleft_15deg.stl	gripper_15deg	5.18 g	0.13 €	0.13 €		PLA
P14_Gripperright_15deg.stl		6.80 g	0.18 €	0.18 €		PLA
P15_HC-SR04_prt1.stl	HC-SR04-holder	5.01 g	0.13 €	0.13 €		PLA
P16_HC-SR04_prt2.stl		8.41 g	0.22 €	0.22 €		PLA
P17_Module1.stl	module 1	15.98 g	0.42 €	1.26 €		PLA
P18_Module2.stl	module 2	15.82 g	0.41 €	0.41 €		PLA
P19_Module3.stl	module 3	11.72 g	0.30 €	0.30 €		PLA
P20_Stamp.stl	stamp	13.91 g	0.36 €	0.36 €		PLA
P21_StampRack.stl	stamp rack	13.45 g	0.35 €	0.35 €		PLA
P22_SupplyStorage.stl	supply & storage	29.31 g	0.76 €	1.52 €		PLA

The amount of filament listed in the table includes all subsequent components printed for the project. If the weight of the individual components is added up, a total of 436 g PLA was used. Extrudr PLA NX-2 (1.75 mm) filament was used on a Bambu Lab X1 Carbon FFF-printer with a 0.4 mm nozzle and standard settings for the slicer. As there are no special requirements for the 3D-printed parts, other PLA or standard filaments can also be used. When selecting the camera as the imaging sensor, it should be ensured that the resolution is sufficient for the distance to the work surface to perceive the change in brightness when the protective foil is removed (see Table 2).

## 5 Build instructions

**Fig. 6:**
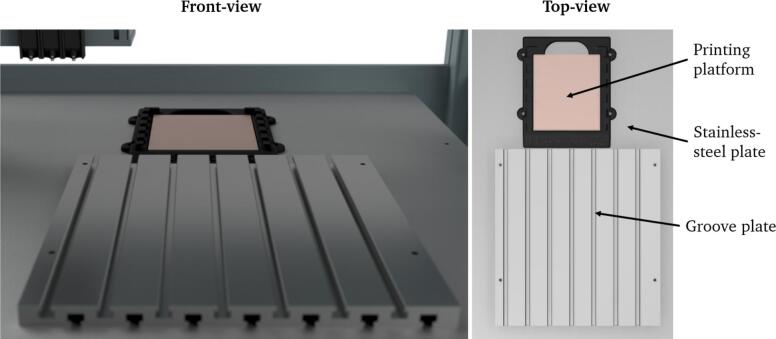
Rendering of the work plate in the cell of the 3D-bioprinting system. The work plate is made of stainless-steel and has a fixed insert for the base plate (P3) and fixed mounting points that can be used, for example, to place a groove plate at a defined location.

An existing 3D-bioprinting system based on a CNC machine serves as the foundation for the developed system. The work area, which allows sterile working due to a stainless-steel surface on the work plate (Fig. 6) and a hood for laminar flow, has fixed mounting points and an insert at the printing platform. A groove plate (C3) as a complete module with all mounted components can be flexibly inserted or removed at a defined location at the mounting points.

This is important as the printer is used by several users in everyday laboratory work and the installation space may be required for different applications. Otherwise, all the individual components would have to be assembled and disassembled repeatedly and the entire system would have to be recalibrated each time it was set up. In addition, the 3D-bioprinter has a defined slot for different modules on the printing platform. Different base plates can be used and fixed here for the respective application.

In defining and selecting specifications for the overall system, a requirement list and a morphological box were used. Detailed design decisions are outlined in the respective steps during assembly.

The steps for setting up the system are chronologically described as follows:

**1.** **Assembly and attachment of the components on the portable groove plate**

**Fig. 7:**
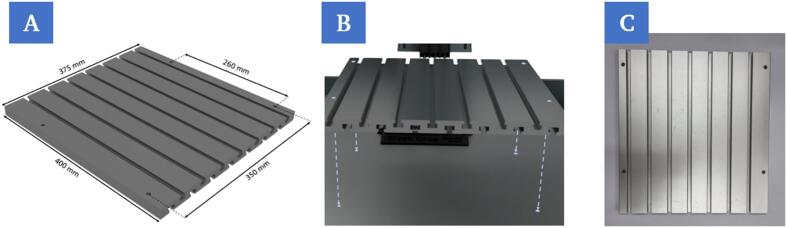
Illustration of the dimensions of the groove plate (A). Insertion of the groove plate onto the work surface of the 3D bioprinter at the designated screw points (B). Empty groove plate without mounted components (C).

**Fig. 8:**
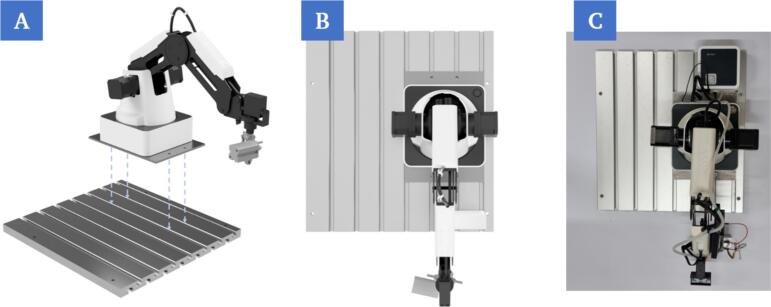
Mounting of the robot on the groove plate using four screw connection points (A). Rendering of the top view of the mounted robot (B). Top view of the mounted robot (C).

**Fig. 9:**
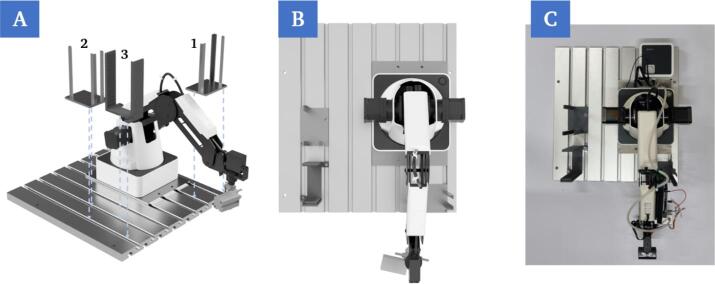
Mounting of the chip supply (1) and storage (2) as well as the cover slip supply (3) on the groove plate using the corresponding screw points (A). Rendering of the top view of the mounted components (B). Top view of the mounted components (C).

The components below (1a – 1c) are fixed to the groove plate (C3) using suitable screws (C4) and sliding blocks (C6). The groove plate used (Fig. 7) itself, after all components are mounted, is later fixed to the worktop using the four screw points and the M6 × 50 mm screws (C5) (Fig. 7B).

a. The robot (C1) is mounted onto the groove plate in the first step (Fig. 8A). The position must be set in such way that sufficient space is available for positioning the other components on the plate, but at the same time the chip holder modules on the printing platform are also within the working range of the robotic arm (Fig. 8).

b. The supply and storage stations (P22, P6) are then mounted (Fig. 9A). These must be positioned in the work area of the robot. Furthermore, it must be ensured that the construction does not cause any collisions in the robot’s path to the printing platform (Fig. 9).

**Fig. 10:**
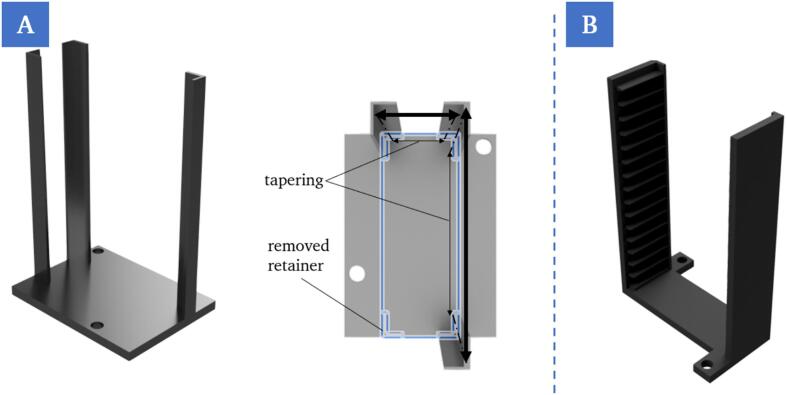
Rendering illustrating the optimized design of the chip supply and storage with the design adjustments. To facilitate vertical accessibility for the robot, one of four retainers was removed. To compensate for inaccuracies when unloading or loading the chips, the remaining three retainers were designed in a way that they are tapered towards the bottom and allow the chip to be locked in position automatically (A). Rendering of the cover slip supply, which enables horizontal removal of single cover slips. As the cover slips are only a few hundred micrometers thick, they cannot be stacked directly on top of each other and removed vertically by the robot (B).

A magazine that can be loaded and unloaded vertically was selected for the chip supply and storage points (P22). Iterative design changes were made to the basic structure (Fig. 10A). This serves to improve process reliability by compensating for repetition inaccuracies of the robot. The cover slips are supplied via a magazine (P6), which can be unloaded horizontally (Fig. 10B).

**Fig. 11:**
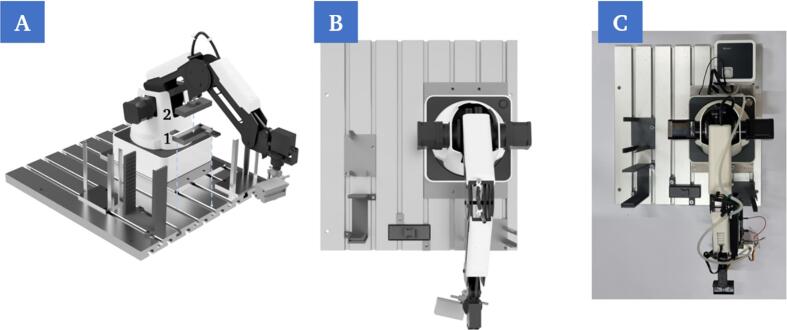
Mounting of the stamp rack (1) on the groove plate using the corresponding screw points and positioning of the stamp (2) in it (A). Rendering of the top view of the fully pre-assembled groove plate with all components (B). Top view of the fully pre-assembled groove plate with all components (C).

Up to 15 chips can be produced in sequence in this configuration before the user has to restock.

c. The stamp rack (P21), in which the stamp (P20) for the fixing of the cover slip is stored, is then mounted (Fig. 11). The positioning and orientation are set to ensure a short path between the stamp holder and the chip holder module.

**2.** **Gripper assembly**

**Fig. 12:**
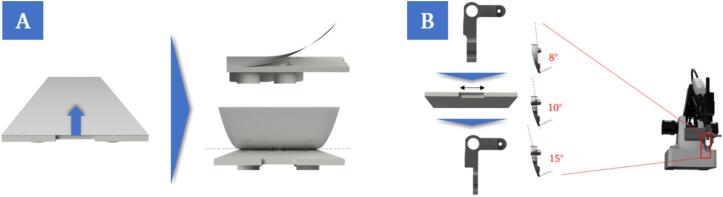
Rendering of the raw chip, which is initially covered by a foil to protect it from contamination. The protective foil needs to be disposed of before filling the reaction chambers. The chip has a small cut-out to facilitate the removal of the foil (A). Illustration of the design modifications to the gripper, exemplified on one gripper finger. The gripper fingers are identical, except for their attachment to the actuators. The width of the gripping surface was reduced to match the cut-out width on the chip. In addition, the angle of the gripping surface has been adjusted. The version with a negative inclination angle of 8° proved to be the most reliable (B).

The design of the gripping system of the robot was optimized for the microfluidic chip used. The chip has a cut-out as a gripping aid for removing the protective foil (Fig. 12A). The width and angle of the gripping surfaces were modified for this purpose (Fig. 12B).

**Fig. 13:**
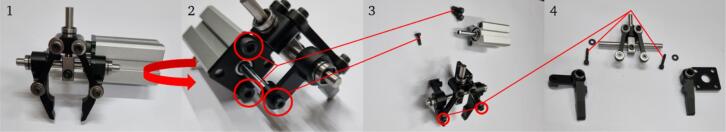
The gripper must first be detached from the robot via the muff coupling (1). Then, by turning it to the side, the marked screw connections on the pneumatic cylinder and cylinder rod must be loosened (2). The screw connections that connect the gripper fingers to the movable holders can now be loosened (3). The gripper can then simply be pushed laterally over the linear guide rail to the outside (4).

To install the optimized gripper, the original gripper must first be removed (Fig. 13). If the correct gripper for the intended application has already been installed, step 3 of the build instructions can be performed immediately.

**Fig. 14:**
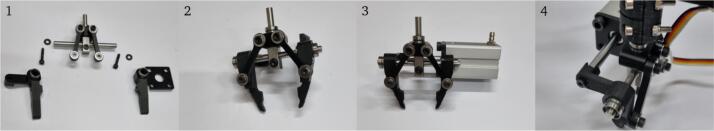
Gripper in disassembled state (1). Slide the selected gripper fingers (gripping surfaces pointing inwards) onto the horizontal linear guide rail via the linear bearings and screw the gripper fingers to the movable holders (2). Attach the gripper to the pneumatic cylinder and cylinder rod via the screw connection points (3). Attachment of the entire gripper to the robot via the muff coupling (4).

The new gripper can then be installed (Fig. 14).

**3.** **Mounting of the sensors**

**Fig. 15:**
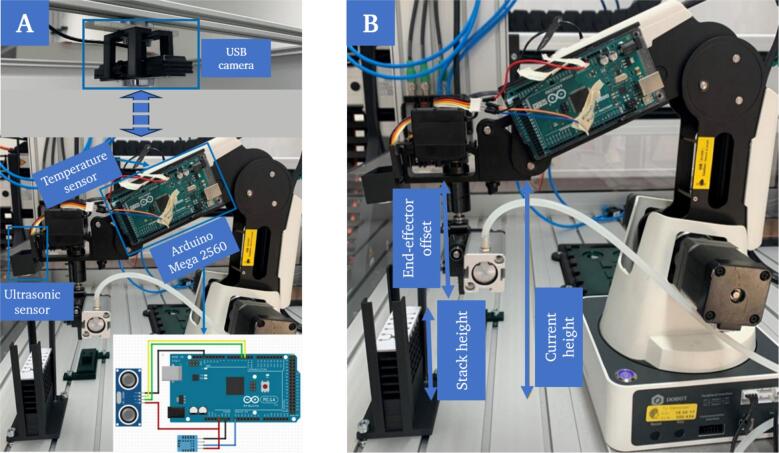
Overview of the mounted sensors (A). The calculation of the distance between the gripper and the object, here for example the top chip of a stack in the chip supply, is calculated by subtracting the end effector offset from the current height and the difference between result and the stack height (B).

The sensors must be installed before the system can be put into operation (Fig. 15A).

a. The ultrasonic sensor (S1) is attached to the center of the robot's forefront with a special mount (P15 – P16). Due to the interchangeable end-effector, the standard reference point is fixed to the structure of the robot. When using a specific tool, the offset to the standard reference point must therefore be specified. The home parameters, which specify the starting position of the end effector, are set so that the end effector does not collide with any object when performing the reference run. The ultrasonic sensor is positioned in such way that the gripper does not interfere with the sensor's field of vision. After attaching the sensor, the end effector offset must be determined once and the current distance from the sensor to the groove plate must be determined as a reference for calibration. After calibration, the distance to any object to be gripped can be determined (Fig. 15B).
b. The temperature sensor (S2) is mounted at the right-hand side of the front arm with a special mount (P7–P8).
c. The microcontroller (C2) is mounted to the left-hand side of the front arm with a special mount (P1–P2).
d. The webcam is attached to a support profile on top of the work area with a universal mount (P4–P5, Fig. 15A).

**4.** **Insertion of the base plate at fixed position in the working area of the 3D-bioprinter**

**Fig. 16:**
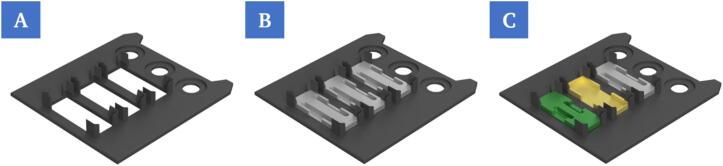
Rendering of the base plate without any modules (A). Rendering of the base plate with the module used in this work (P17) (B). Rendering of the base plate with three different modules (P17, P18, P19) (C).

The base plate (P3) used here has a modular structure (Fig. 16). It has 78.1 × 30.1 mm cutouts in which various modules can be inserted, such as chip holders to lock the chips in place for printing.

Modules with specific properties can be used as chip holders, for example made of thermally conductive material to ensure the heating or cooling of chips.

**Fig. 17:**
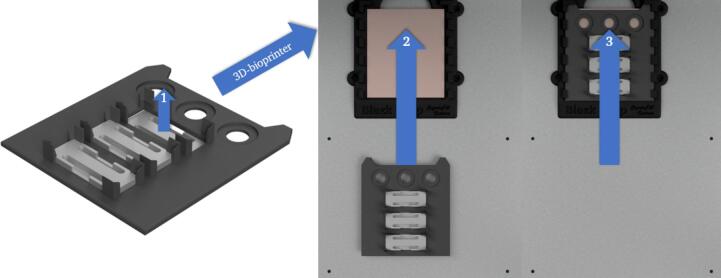
Fitting of the modules into the base plate 1. Directed insertion of the base plate with modules into the fixed slot on the printing platform (2, 3).

Prior to the insertion of the pre-assembled groove plate module into the working area, the base plate with suitable modules (1) must be slid into the designated fixture on the printing platform (2) – (3) (Fig. 17).

5. Insertion and mounting of the groove plate at fixed position in the work cell of the 3D-bioprinter

**Fig. 18:**
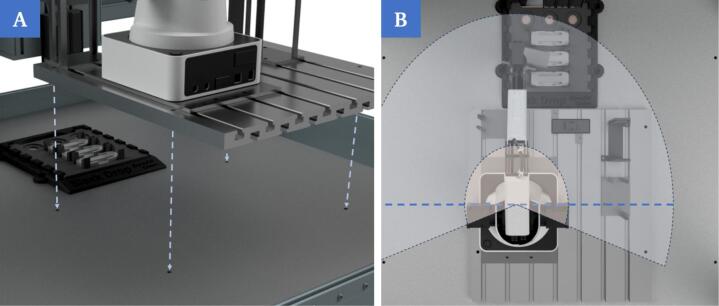
Insertion of the pre-assembled groove plate with all components via the fixed mounting points (A). Rendering of the complete setup in the working area of the bioprinter. All components are within reach of the robotic arm (B).

After all the steps above have been executed in sequence, the groove plate with all mounted components can be positioned in the working area and screwed into place (Fig. 18A) to setup the whole build for operation (Fig. 18B). Beforehand, it should be checked that the correct base plate has been inserted on the printing platform and that all components on the groove plate are in the correct position.

After use, the entire groove plate can simply be removed to free up the worktop of the bioprinter for other applications. When reusing the plate in the same configuration, steps 1–3 can be skipped and only steps 4 and 5 need to be carried out.

When setting up the system, it must be ensured that the user beforehand has received safety training for the specific laboratory and has been instructed in the handling of the machines used. All electrical devices in the cell – especially the 3D-bioprinter and robot – should be switched off or disconnected from the power supply at the time of assembly. Careful handling is important when mounting the components on the groove plate and inserting the plate into the work area, e.g., to avoid contusions or other physical injuries. Furthermore, there is no particular safety hazard.

## 6 Operation instructions

Once the system has been set up according to the steps in the build instructions, it can be taken into operation. The following steps should be adhered to strictly and in sequence to ensure proper performance:

1. Check that the assembly is set up completely according to the build instructions.
2. Connect the robot to the power supply and PC.
3. Switch the robot on and start the software.
4. Test the working range of the robot (can all stations be approached without collision or error by an end stop). Change the setup if necessary and return to step 1.
5. Test the functionality of the sensor system.
6. Calibration according to the current configuration (only necessary if something was changed to the configuration after the last use).
7. Defining and entering the fixed points in the configuration file.
8. Start the 3D-bioprinter and set the required settings for the intended process.
9. Make sure that all connections are established properly and are recognized by the central processing unit (robot including sensors, 3D-bioprinter, camera).
10. Initialize the virtual interface.
11. Run a test cycle and return to step 6 if necessary.
12. (Re-)Stock the chip and cover slip supply.
13. Fill the cartridge(s) of the 3D-bioprinter with the required bio-ink(s).
14. Start the process.
15. When all chips have been processed, return to step 12. If no new run is to be started, proceed to step 16.
16. Shut down all systems after the process is finished before you start the disassembly.

During operation, it must be ensured beforehand that the user has received safety training for the specific laboratory and has been instructed in the handling of the machines and materials used. The user should be permanently present during the process and monitor the process in the event of errors to be able to intervene. Furthermore, there is no particular safety hazard, since the entire system is embedded in a safety cell and the forces that can be applied by the educational robot are low. An emergency stop is triggered in the event of collisions to stop the system.

## 7 Validation and characterization

As already outlined, automation and standardization in the production of OoCs holds great potential for overcoming current hurdles on the way towards achieving the technology's economic potential (chapter 1). The approach described here addresses this by using a single 4-axis-robot, basic sensors and electronics as well as 3D-printed parts. Furthermore, the user does not need any special prior experience in handling robotics or programming controllers.

### 7.1 Identification and evaluation of process errors

The individual process steps in the manipulation and transportation of the components have different failure potentials. For this reason, preliminary tests were carried out in which the individual process steps were examined and evaluated with regard to their vulnerability to errors. For the tests 12 “dry” runs (video1.mp4) were carried out to determine which process steps have the greatest potential for errors. The process chain is divided into steps before and after printing (Table 3). The actual printing process was left out of the evaluation and only the manipulation and transport steps were considered, as this work explicitly addresses the bridging of manual intermediate steps and not the 3D-bioprinting process. The printing process is a complex task in its own and is addressed in other studies. The evaluation was performed in binary form, i.e. the respective process step can only be successful or unsuccessful and a qualitative evaluation of the steps was omitted. A step is considered unsuccessful if the process has to be aborted and cannot be continued. If the process was aborted, a prepared chip was added again at the respective position in order to obtain the same number of samples for every process step.

**Table 3:** Overview of the success rates for the individual process steps with a sample size of 12 runs per step (n = 12). The steps are categorized into a pre-printing and post-printing phase. The post-printing phase proved to be robust and showed no errors. In the pre-printing phase, errors were detected during chip pick-up and removal of the protective foil.

	**Steps of the process chain**						
	**Pre-printing**			**Post-printing**			
	Pick up raw OoC	Place on bioprinter	Remove protective foil	Pick sealing up	Place & fix sealing	Pick up filled & sealed OoC	Place on storage
Success rate in %	83	100	67	100	100	100	100

While the transportation to the printing area and subsequent preparation for the printing process of the chips led to errors, the post-processing and storage of the chips were reliable. The sample size during the tests was small and it must be expected that errors can potentially occur in all process steps.

The errors during chip pick-up are due to insufficient tolerances in the first iteration of the supply or insufficient repetition accuracy of the robot. The error was subsequently corrected by adjusting the geometry of the supply (Fig. 10A). Another solution, which offers further flexibility but is significantly more complex to implement, is the integration of a force-torque sensor and an associated control system. However, this was not considered for reasons of cost and complexity.

**Fig. 19:**
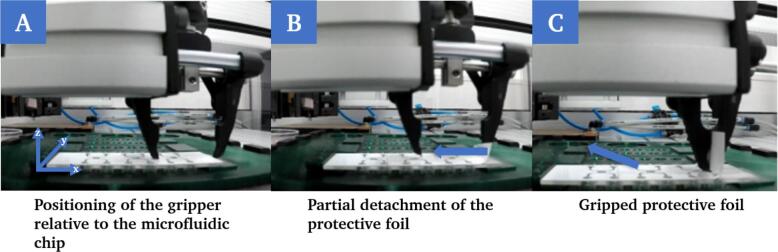
After the parallel gripper has been positioned centrally relative to the chip at the cut-out height so that it opens and closes along the negative x-axis (A), partial removal of the protective foil begins by closing the gripper (B). The robot then moves along the negative x-axis and, from the last third of the chip length, also performs a movement in the positive z-direction until the foil is completely removed (C).

Removing the protective foil from the chip (Fig. 19) shows the greatest potential for error (Table 3).

**Fig. 20:**
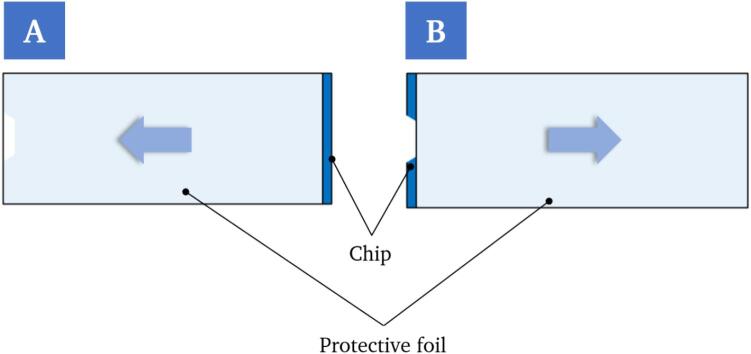
Illustration of extreme cases of positioning of the protective foil on the chip. In case 1, the protective foil protrudes to the edge of the chip beyond the cut-out (A). In case two, the protective foil is shifted in the opposite direction as far as possible (relative to the length of the chip) with no part of the foil protruding beyond the cut-out.

The critical aspect of this process step is the partial detachment of the protective foil. If this part is not successful, the protective foil cannot be gripped and completely removed by the robot. If the protective foil is not completely removed, it is not possible to seal the chip with the cover slip at a later stage and the chip will be unusable. In this case, the printing process can also be hindered, meaning that the printer cannot print in the intended chambers. In the experiments, the positioning of the protective foil was identified as the main reason for errors in this process step. Two cases occur (Fig. 20), which can influence the gripping of the protective foil. The positioning of the protective foil, thus the extreme cases, result from tolerances in the production of the raw chips.

Case 1 (Fig. 20A) was initially seen as beneficial, as a larger part of the protective foil sticks out of the pocket. Contrary to expectations, this case presents a challenge, as the gripper grips both the protective foil and the adhesive layer underneath and it is not possible to remove the protective foil alone. In the second case (Fig. 20B), no part of the protective foil protrudes beyond the pocket and the gripper is unable to grasp it. Assuming a normal distribution of the positioning of the protective foil, however, the cases mentioned represent extreme cases.

**Fig. 21:**
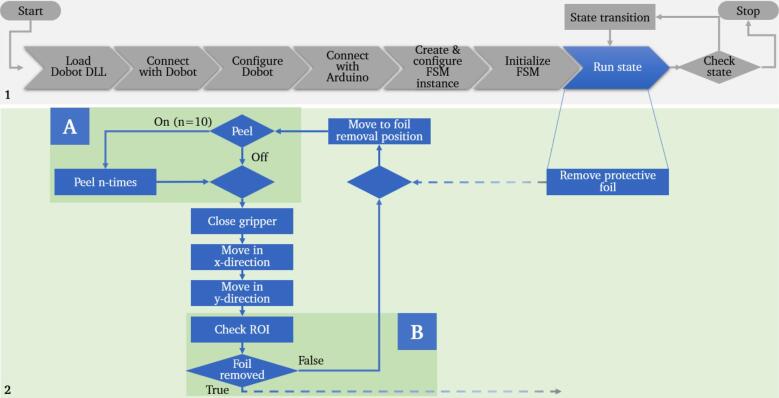
Simplified flowchart of the algorithm for the removal of the protective foil with a function for repeated peeling at the edge of the foil (A) and a monitoring mechanism using a camera (B) to reduce the error rate in this process step.

To minimize the likelihood of errors during partial detachment, gripping and removal of the foil, regardless of its positioning, preventive measures were taken. On the one hand, the gripper geometry was adapted according to the chip design (Fig. 12). In addition, a function that can be switched on and off was integrated into the algorithm, which allows repeated peeling by moving the gripper horizontally along the edge of the protective foil. This mechanism is time-consuming but increases the success rate and can be implemented without further design adjustments to the system (Fig. 21A). In addition, a simple web camera was installed vertically above the working area. This camera monitors a defined area of the chips placed on the modules for printing. The detected brightness values in the ROI are used to verify whether the foil has been successfully removed after the robot has finished the peeling movement (Fig. 21B). If the step is not successful, the foil removal step is repeated.

**Fig. 22:**
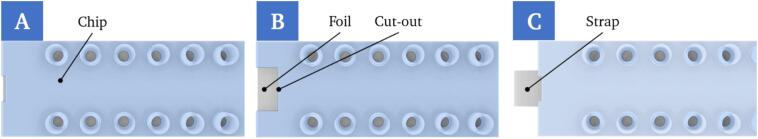
Rendering of a chip with the protective foil as originally supplied, viewed from the bottom (A). Rendering of a customized chip with enlarged cut-out (B). Rendering of a chip with customized foil with additional strap (C).

Another way to minimize the error rate during removal of the protective foil, which was not pursued further in this work as an existing chip design (Fig. 22A) was used, is the simple adaptation of the chips. One solution involves adapting the design of the cut-out on the edge of the chip as a gripping aid. The cut-out can be enlarged, and the foil should not adhere in this area (Fig. 22B). Another conceivable solution is a strap at the edge of the protective foil, that the gripper can easily grasp (Fig. 22C).

### 7.2 Validation of the process chain

**Fig. 23:**
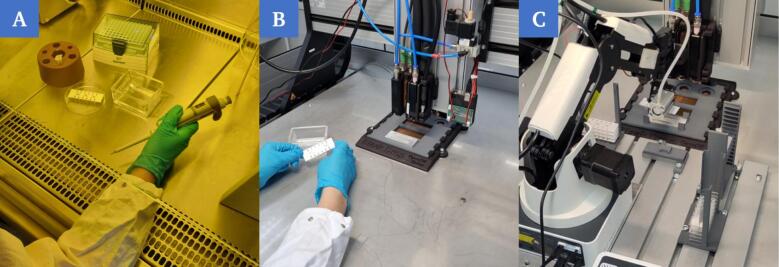
Completely manual manufacturing process of the OoC by trained laboratory personnel (A). Semi-automated manufacturing process of the OoC by using a 3D-bioprinter and manual intervention of the user to manipulate the chips used (B). Automated manufacturing process of the OoC by using a 3D-bioprinter and bypassing manual handling steps by using a robot and peripheral structures (C).

Potential errors were identified in the preliminary tests and corresponding safety mechanisms were integrated to improve process reliability (chapter 7.1). The focus was placed on the process steps that were identified as error-prone (step 1 and step 3). The optimized system and the developed process chain can be validated in terms of their functionality and performance. Three scenarios, regarding the handling adjacent to the printing process, were compared for this purpose (Fig. 23).

The result for manual handling of the chips and pipetting of the cell-laden bioinks (Fig. 23A, video2.mp4) is individual and variable during repetition of the process, even with experienced and trained personnel. More complex models, that may require very small volumes of droplets in a certain geometric arrangement, cannot be realized as the minimum volume that can be achieved with a pipette is in the range of 0.5–1 µl, depending on the material, as well as due to a lack of repeatability by the user. In the second scenario, the process is semi-automated (Fig. 23B). This paper explicitly addresses handling and manipulation in adjacent phases and not the printing process itself. However, handling and the printing process have fixed interfaces and may be categorized as a whole. With regard to the overall process, this scenario is therefore labeled as “semi-automated” in order to differentiate it from the completely manual handling and pipetting in the first scenario. The transport and manipulation steps are still carried out manually, but the 3D-bioprinter dispenses the bioink (video3.mp4). By using the 3D-bioprinter, a repeatable result is achieved when applying the bioink. Nevertheless, there is still further potential for optimization here, as the user must actively intervene in the process at short intervals, which also makes sterile work more difficult. The third scenario (Fig. 23C) comprises the setup developed in this work and the associated process steps. A certain number of chips and cover slips are placed in the respective supply stations and a repeatable process is started. A maximum of 15 chips can be processed in sequence in the setup shown. The process – starting with pre-loaded supply stations with a certain number of microfluidic chips and cover slips and ending with filled OoC devices ready for perfusion – is automated and requires no manual intervention by the user (video4.mp4). This manufacturing process proves to be fast, repeatable, and reliable. The process could be further accelerated in the future by parallelizing manipulation and printing.

In addition to the time savings, the automation of the handling steps offers further advantages. No explicitly trained personnel with laboratory experience is required and there is no need for manual intervention in the process. As a result, the entire system can be integrated in a sterile environment and the process produces repeatable and precise results. After processing, the manufactured OoC devices must be manually transferred to their long-term cultivation environment and for dynamic assays, manually connected to a perfusion system.

#### 7.2.1 Manufacturing scaling and speed comparison

The automation of the considered process should bring advantages, e.g. in terms of standardized workflows or potential time gained, compared to the manual approach. In order to quantify time expenditure and possible work potential, these must first be recorded. Several time factors are taken into account, including setup time $\left(t_{s}\right)$ production time $\left(t_{p}\right)$, handling time $\left(t_{h}\right)$, automated time $\left(t_{a}\right)$ and operator attendance time $\left(t_{o}\right)$ (Table 4). A completely manual procedure for producing the OoC devices (Fig. 23A) is not included in the analysis. Accordingly, times for manual handling (Fig. 23B) in combination with 3D-bioprinting $\left(P_{1}\right)$ as well as the use of the presented system for robotic handling for process phases adjacent to 3D-bioprinting (Fig. 23C) after initial setup $\left(P_{2}\right)$ and further use for a determined setup $\left(P_{3}\right)$ are included.

**Table 4:** Time factors recorded to quantify the influence of automation on the production process.

	Setup time $\left(t_{s}\right)$	Production time $\left(\overset{¯}{t_{p}}\right)$	Handling time $\left(\overset{¯}{t_{h}}\right)$	Automated time $\left(\overset{¯}{t_{a}}\right)$	Operator attendance time $\left(t_{o}\right)$
Manual handling $\left(P_{1}\right)$	10.5 min	91.9 s	66.9 s	0 s	33.5 min
Automated after initial setup $\left(P_{2}\right)$	14.7 min	86.6 s	61.6 s	$t_{a}=t_{h}$	35.3 min
Automated for further use $\left(P_{3}\right)$	29 min	86.6 s	61.6 s	$t_{a}=t_{h}$	21 min

The setup time comprises the preparation of the 3D-bioprinter (booting up the system, setting print parameters, filling the cartridge with bioink and loading the print model) and additionally, for $P_{2}$ and $P_{3}$, the respective steps described in sections 5 and 6. After initial setup for a specific configuration, the pre-assembled groove plate can be inserted into the printer and installation and calibration steps can be skipped, which reduces the setup time for $P_{3}$ substantially compared to $P_{2}$. The production time $t_{p}$ consists of the individual handling time $\left(t_{h}\right)$ and, in the presented example a printing duration of 25 s, which is identical for all three processes for the selected print model. The values for $\overset{¯}{t_{p}}$ and $\overset{¯}{t_{h}}$ refer to the average time required per chip. In the presented configuration for $P_{2}$ and $P_{3}$, 15 chips can be processed in sequence without manual intervention. To determine the average values $\overset{¯}{t_{p}}$ and $\overset{¯}{t_{h}}$, 15 chips were therefore processed for each, the manual handling $(P_{1}$) and the automated handling $\left(P_{2},P_{3}\right)$. The operator attendance time $t_{o}$ describes the sum of all times during which the operator is unable to perform other laboratory tasks.

**Fig. 24:**
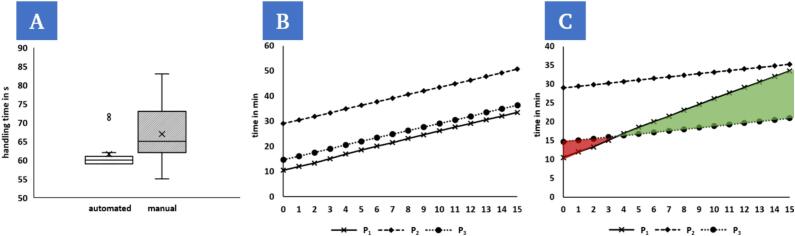
The automated approach shows a lower average handling time per chip $\overset{¯}{t_{h}}$ for the examined samples (n = 15) and a lower variation of times compared to the manual procedure (A). The total time $t_{T}$ for manufacturing a certain number of chips depends strongly on the setup time, making the manual process the fastest overall, even after processing 15 chips (B). Operator attendance times $t_{o}$ (C) can be taken into account as an indirect factor on productivity. With an existing configuration, the automated process shows a time advantage from the fourth chip onwards and the setup time becomes progressively less important as the number of chips processed increases.

If we compare the values for the average handling time per chip $\overset{¯}{t_{h}}$ (Fig. 24A), the average for the observed samples n=15 is slightly lower for the automated procedure compared to the manual approach. Taking the influence of the user's individual experience into account, the value for the manual procedure can fluctuate greatly. Overall, it can be observed that the variance for the automated process is significantly lower, and $t_{h}$ fluctuates considerably less. Outliers in the automated process are due to errors in the removal of the protective foil and the repetition of this process step. The removal of the protective foil also has the greatest influence on the time fluctuations in the manual process.

The calculation of the total time $t_{T}$ is based on the sum of the setup time and the sum of all production times:

$$t_{T}=t_{s}+\sum_{i=1}^{n}t_{\mathrm{pi}}$$

whereas n corresponds to the number of chips per cycle (Fig. 24B). Considering only the value $t_{T}$, the lowest total time is found for the manual procedure after processing 15 chips. For the manual process $\left(P_{1}\right)$, $t_{T}=33.5min$ and for the automated process, $t_{T}=50.7min$ $\left(P_{2}\right)$ and $t_{T}=36.4min$ $\left(P_{3}\right)$ respectively. Due to the large differences for $t_{s}$ and the small deviation for $\overset{¯}{t_{h}}$, the pure loss of time per process cycle cannot be compensated for by automation in the presented configuration.

However, since the operator does not have to intervene in the process for the time of the automated handling, it is useful to calculate the required operator attendance time $t_{o}$. It is calculated from the difference between the total time $t_{T}$ and the sum of all automated times $t_{\mathrm{ai}}$:

$$t_{o}=t_{T}-\sum_{i=1}^{n}t_{\mathrm{ai}}$$

For the manual approach, $t_{o}=t_{T}$, as the operator is involved in the process for the entire handling time and $\sum_{i=1}^{n}t_{\mathrm{ai}}=0$. In the automated approach, $t_{a}=t_{h}$, so that the entire handling time for the operator is eliminated. By plotting the time against the number of chips (Fig. 24C), a break-even point can be determined from which the automated approach offers a time advantage. The reduced need for the operator to be present results in an advantage, as the operator can, for example, prepare the next test series or perform other laboratory tasks in the meantime. In theory, there would also be a time advantage for $P_{2}$ over $P_{1}$ from the 16th chip onwards. Since setup times for further test series could be parallelized with the process, which could only be completed after the process sequence in the manual approach, a time advantage would also arise here from a subsequent test series. The automated time $t_{a}$ in the analysis here only refers to the handling addressed in the work and does not include the automated printing time for both approaches.

The gained laboratory time $t_{l}$ resulting from automation for the operator is calculated from the difference between the operator attendance times for the automated procedure $t_{o,auto}$ and the manual procedure $t_{o,man}$.

$$t_{l}=t_{o,auto}-t_{o,man}$$

An advantage from the automated approach can be assumed in the case of $t_{l}>0$. When evaluating the results, it must be taken into account that they were recorded by a single test person and that a larger number of samples is required for further validation. Nevertheless, the demonstrator presented shows the potential of automating the process chain for the production of OoCs. The presented solution offers the capability to produce 15 chips without manual intervention – from an existing substrate to a perfusion-ready OoC device – and enables the efficient production of larger samples in research laboratories for the statistical validation of research results.

#### 7.2.2 Comparison of leak tightness for perfusion

The reliable and tight sealing of the chips is essential for the subsequent perfusion and thus the usability of these models. The adhesive bonding of the cover slip to the surface of the chip has proven to be a reliable mechanism for this.

**Fig. 25:**
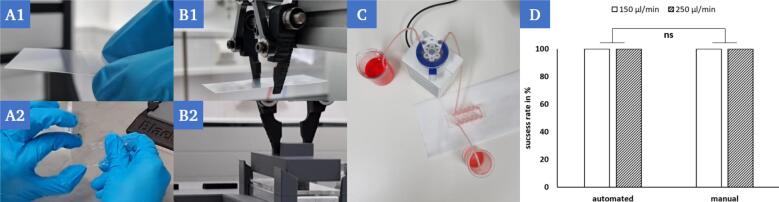
Manual transfer of the cover slip to the chip (A1). Fixation of the cover slip by manual pressing (A2). Robotic transfer of the cover slip to the chip (B1). Fixation of the cover slip via robotic handling of the stamp (B2). Perfusion through a peristaltic pump of a filled and sealed chip with red colored water on white filter paper to determine the leak tightness (C). The way of handling when sealing the chips has no significant (ns) influence on the leak tightness during subsequent perfusion for different flow rates [150; 250] µl/min (D). (For interpretation of the references to color in this figure legend, the reader is referred to the web version of this article.)

To exclude leaks caused by the sealing procedure, chips were sealed manually by a test person and robotically automated to assess and compare the leak tightness. Prior to sealing, the reaction chambers of the chips were filled with 2.2 µl hydrogel (0.5 % agarose) each. The agarose (low gelling temperature, Sigma-Aldrich) was pipetted with an Eppendorf Research plus pipette (0.5–10 µl) at a temperature of 37 °C. The chips were then sealed manually (Fig. 25A) and automated (Fig. 25B), respectively. The samples were then stored at 4 °C for 15 min to ensure gelation of the agarose. After sealing of the chips and gelation of the hydrogel, the channels of the chips adjacent to the reaction chambers were connected to a peristaltic pump (medorex micro-pump HP) via the corresponding Luer connectors (Fig. 25C) and flushed with red-colored water at a set flow rate of [150; 250] µl/min for 15 min. The sealed and perfused chips were placed on absorbent filter paper for the entire duration of the experiment and the observer then visually assessed the leak tightness in binary form.

Two scenarios (automated and manual) were evaluated using n = 3 samples per scenario and set flow rate with binary outcomes 'success' and 'failure' determined by the observer. The success rate was then calculated from this. In both scenarios, a success rate of 100 % was determined for all set flow rates (Fig. 25D).

The observation of a 100 % success rate in either scenario indicates that under the conditions tested, both settings are suitable, and a tight seal can be guaranteed. The fabricated OoC devices can resist perfusion and therefore allow the simulation of a physiologically inspired environment in the model.

### 7.3 Final assessment of the process chain and the overall system

In this work, an open source lab-automation solution for the standardized fabrication of perfusion-ready OoC devices utilizing 3D-bioprinting and an educational robot was implemented. For this purpose, a suitable process chain was first developed, weaknesses in it were identified and appropriate countermeasures were taken to overcome errors as well as mechanisms integrated to implement these. Affordable components were used in the development and realization of the system in order to create low-threshold access, in contrast to conventional expensive laboratory automation. The system developed was created as a laboratory demonstrator and, so far the only available in this form, represents an important basis for further work on scaling up OoC manufacturing processes.

The following list summarizes the most important capabilities, limitations and potentials of the system:

• The process chain enables the repeatable production of perfusion-ready OoC devices, and the entire system can be integrated into a closed sterile environment.
• The system has been evaluated positively on a laboratory scale and represents a valuable tool on the way to scaling-up and standardizing the industrial production of OoCs.
• Acquisition costs are comparatively low in the context of lab automation and the openness of the system makes it easy to expand and customize.
• The selected basic structure of the underlying algorithm, given by the FSM, is in line with the standards defined by the SiLA consortium and has a defined number of states that can be extended.
• In the evaluation and validation of the system, it was shown that the automation of the process for producing perfusion-ready OoC devices offers the advantage of being able to be integrated into a sterile environment and also holds great potential in terms of speed, accuracy and standardization.
• For the used chip design, the removal of the protective foil poses the greatest potential for error. Although this can be reduced by adjusting the algorithm and monitoring the process step with a camera, it still remains the biggest weakness in the workflow. A future adaptation of the chip design by the manufacturer, which facilitates the robotic gripping of the foil, could help to make this potential error obsolete.
• Calibration is necessary for the initial setup or after modifications to the setup and there is currently no plug-and-play solution available.
• The process still requires the supervision of a responsible person who can intervene in an emergency and is not fully automated in the sense that a confirmation in the printer control software is required to start the printing process.
• Extending the system by adding a force-torque sensor with the associated control system can further reduce the vulnerability to errors during pick and place tasks and improve overall process reliability.
• To further increase flexibility and additionally parallelize process steps, tracking systems or linear axes can be integrated in the future. Furthermore, the creation of an interface and direct connection to an autonomous module for cultivation and assaying as well as automated imaging [22] could offer additional success.

### CRediT authorship contribution statement

**Nils Lindner:** Writing – original draft, Visualization, Validation, Supervision, Methodology, Data curation, Conceptualization. **Andres Mejia-Wille:** Validation, Software, Investigation. **Anna Fritschen:** Writing – review & editing. **Andreas Blaeser:** Writing – review & editing, Project administration, Conceptualization.

## Declaration of competing interest

The authors declare that they have no known competing financial interests or personal relationships that could have appeared to influence the work reported in this paper.

## Footnotes

1 Bioprinting is a digital biofabrication process used to produce complex and heterogeneous 3D cell culture systems and living tissues. This method utilizes the principles of additive manufacturing to replicate the macroscopic anatomy of the target tissue by printing living cells in a hydrogel matrix layer by layer. The printed precursor structures are cultivated in specific bioreactors, on-chip platforms or host organisms to mature and develop into biofunctional tissue units. [20].

## References

[1] Ramadan Q., Zourob M. (Jul. 2020). Organ-on-a-chip engineering: Toward bridging the gap between lab and industry. Biomicrofluidics.

[2] V. Allwardt et al., Translational roadmap for the organs-on-a-chip industry toward broad adoption, Bioeng. 2020, 7 (3) 112, 2020, doi: 10.3390/BIOENGINEERING7030112. 10.3390/bioengineering7030112 PMC7552662 32947816

[3] Sosa-Hernández J.E. (Oct. 2018). Organs-on-a-chip module: a review from the development and applications perspective. Micromachines.

[4] Fritschen A., Blaeser A. (Jan. 2021). Biosynthetic, biomimetic, and self-assembled vascularized Organ-on-a-Chip systems. Biomaterials.

[5] Q. Wu et al., Organ-on-a-chip: recent breakthroughs and future prospects, Biomed. Eng. OnLine 2020 191 (1) 1–19, 2020, doi: 10.1186/S12938-020-0752-0. 10.1186/s12938-020-0752-0 PMC7017614 32050989

[6] Ma C., Peng Y., Li H., Chen W. (2021). Organ-on-a-chip: a new paradigm for drug development. Trends Pharmacol. Sci..

[7] Sung J.H. (2019). Recent advances in body-on-a-chip systems. Anal. Chem..

[8] M. Rothbauer et al., A Decade of Organs-on-a-Chip Emulating Human Physiology at the Microscale: A Critical Status Report on Progress in Toxicology and Pharmacology, Micromachines (2021) 12 (5) 470. doi: 10.3390/MI12050470. 10.3390/mi12050470 PMC8143089 33919242

[9] Selimović Š., Dokmeci M.R., Khademhosseini A. (Oct. 2013). Organs-on-a-chip for drug discovery. Curr. Opin. Pharmacol..

[10] D. E. Ingber, Human organs-on-chips for disease modelling, drug development and personalized medicine, Nat. Rev. Genet. 238, 23, 8, 467–491, Mar. 2022, doi: 10.1038/s41576-022-00466-9. 10.1038/s41576-022-00466-9 PMC8951665 35338360

[11] Ingber D.E. (2020). Is it time for reviewer 3 to request human organ chip experiments instead of animal validation studies?. Adv. Sci..

[12] Singh D., Mathur A., Arora S., Roy S., Mahindroo N. (2022). Journey of organ on a chip technology and its role in future healthcare scenario. Appl. Surf. Sci. Adv..

[13] Li R., Loc, Zhang B., Radisic M. (2017). Organ-on-a-chip devices advance to market. Lab Chip.

[14] “Organ-On-Chip (OOC) Market Size is USD 303.6 Million by 2026 at CAGR 39.9% | Valuates Reports,” Valuates Reports, 2021. https://www.prnewswire.com/in/news-releases/organ-on-chip-ooc-market-size-is-usd-303-6-million-by-2026-at-cagr-39-9-valuates-reports-856995001.html (accessed Jan. 04, 2024).

[15] Lindner N., Blaeser A. (May 2022). Scalable biofabrication: A perspective on the current state and future potentials of process automation in 3D-bioprinting applications. Front. Bioeng. Biotechnol..

[16] Probst C., Schneider S., Loskill P. (Jun. 2018). High-throughput organ-on-a-chip systems: Current status and remaining challenges. Curr. Opin. Biomed. Eng..

[17] T. Chapman, “Lab automation and robotics: Automation on the move,” Nat. 2003 4216923, vol. 421, no. 6923, pp. 661–663, Feb. 2003, doi: 10.1038/421661a. 10.1038/421661a 12571603

[18] Hawker C.D. (Dec. 2007). Laboratory automation: total and subtotal. Clin. Lab. Med..

[19] Archetti C., Montanelli A., Finazzi D., Caimi L., Garrafa E. (2017). Clinical laboratory automation: A case study. J. Public Health Res..

[20] D. F. Duarte Campos and A. Blaeser, “3D-Bioprinting,” pp. 201–232, 2021, doi: 10.1007/978-3-030-66749-8_9.

[21] DOBOT, “DOBOT Magician | An all-in-one STEAM Education Platform,” 2022. https://www.dobot-robots.com/products/education/magician.html (accessed Jan. 04, 2024).

[22] Cliffe F.E. (2023). Mera: A scalable high throughput automated micro-physiological system. SLAS Technol..

